# Plasma Neurodegenerative Biomarkers in Cognitively Preserved Nonagenarians

**DOI:** 10.14336/AD.2024.1260

**Published:** 2025-10-30

**Authors:** Estrella Gómez-Tortosa, Pablo Agüero-Rabes, Alicia Ruiz-González, Sonia Wagner, Raquel Téllez, Ignacio Mahillo, Andrea Ruiz-Calvo, María José Sainz, Anna Lena Nystrom, Lucía Cremades-Jimeno, Teodoro del Ser, Pascual Sánchez-Juan

**Affiliations:** ^1^Department of Neurology, Fundación Jiménez Díaz, Madrid, Spain.; ^2^Instituto de Investigación Sanitaria, Fundación Jiménez Díaz (IIS-FJD), Madrid, Spain.; ^3^Alzheimer’s Centre Reina Sofía-CIEN Foundation. Instituto de Salud Carlos III, Madrid, Spain.; ^4^Department of Immunology, Fundación Jiménez Díaz, Madrid, Spain.; ^5^Department of Biostatistics and Epidemiology, Fundación Jiménez Díaz, Madrid, Spain.

**Keywords:** plasma biomarkers, single molecule array, nonagenarians, Alzheimer’s disease;, neurofilament light chain, glial fibrillary astrocytic protein

## Abstract

Plasma biomarkers represent promising tools for the screening and diagnosis of patients with neurodegenerative conditions. However, it is crucial to account for the effects of aging on biomarker profiles, especially in the oldest segments of the population. Additionally, biomarkers in this sample can offer *in vivo* insights into the physiological mechanisms underlying brain aging while concomitantly supporting cognitive preservation. In this study we analyzed plasma Alzheimer’s disease (AD) core biomarkers, neurofilament light chain (NfL), and glial fibrillary acid protein (GFAP) using the Single Molecule Array (SIMOA) platform in 75 cognitively preserved nonagenarians, and compared with baseline samples of 153 volunteers who were cognitively unimpaired (CU) during six years (classified in ≤ 70, and 71 to 85 years of age), and with 108 AD patients. Nonagenarians almost lack the APOEε4 allele, and had significantly higher Aß40, Aß42, p-tau181, NfL, and GFAP, along with a significantly lower Aß42/40 ratio (*P*<0.001) compared with the two CU groups. NfL and GFAP tripled concentrations in nonagenarians. No differences were noted in any plasma biomarker between the younger and older CU groups. Biomarkers correlated strongly with age only when analyzing together CU controls and nonagenarians. Compared with AD cases, nonagenarians showed lower p-tau181 (*P*=0.001), higher total tau (*P*=0.02), and much higher Aß40, Aß42 and NfL levels (*P*<0.001). The levels of GFAP in nonagenarians were similar to those observed in AD patients. In conclusion, cognitively preserved nonagenarians do not develop the AD biomarker signature and exhibit higher levels of Aß42. However, their threefold increase in NfL and GFAP supports their aging brains are somehow resilient to neurodegeneration. These data support caution in the prognosis of clinical dementia based on NfL and GFAP values. Overall, plasma biomarkers in CU individuals remained quite stable till the eighties.

## INTRODUCTION

In the past decade, fluid biomarkers analysis has emerged as a robust tool for the diagnosis of neurodegenerative conditions, in particular of Alzheimer’s disease (AD). AD core biomarkers in cerebrospinal fluid (CSF) have been standardized to support the Amyoid-Tau-Neurodegeneration (A-T-N) scheme for the biological diagnosis of AD [1], with similar performance than amyloid positron emission tomography (PET) [2]. Decreased CSF Aß42 and Aß42/40 ratio together with increased p-tau181 are a specific clinical signature of underlying AD pathology, supporting distinction from other dementia disorders with high sensitivity and specificity [3, 4]. Recent research employing ultrasensitive technologies, such as the Single Molecule Array (SIMOA) platform, has highlighted the potential of parallel plasma/serum biomarkers, which are more accessible, less time-consuming, and cost-effective [5,6]. AD plasma biomarkers exhibit strong correlations with CSF biomarkers and amyloid PET imaging in several studies [7,8].

Neurofilament light chain (NfL) and glial fibrillary acid protein (GFAP), which serve as fluid biomarkers for neuronal injury and astrocyte reactivity, respectively, are less specific to particular pathologies. They have shown different profiles when contrasting AD with frontotemporal dementias (FTD) [7, 9, 10], although the discriminatory power of these biomarkers in clinical populations is limited [11]. NfL concentrations are non-specific to amyloid pathology and increase in non-AD degeneration, particularly in amyotrophic lateral sclerosis (ALS) and in FTD, as well as in cases with secondary neuronal damage [7,12]. Conversely, GFAP is selectively elevated in AD cases compared to FTD and control groups [7,13]. It is recognized as an early marker associated with β-amyloid plaques [13] and as a reliable surrogate of the progression of AD pathology [14].

The validation of biomarkers in blood samples allows for wider population-based studies with potential to predict dementia risk, and also to study individuals within older age-ranges in which lumbar puncture for research purposes has been traditionally avoided. Elevation of both NfL and GFAP plasma levels have been linked to an increased risk of dementia in cognitively preserved individuals, as well as in patients with subjective memory deficits or mild cognitive impairment [15-19]. Nevertheless, NfL is characterized by a well-documented age dependency [20]. Similarly, GFAP levels appear to rise with age in cognitively unimpaired (CU) individuals, independent of Aβ status [21]. Consequently, the predictive and discriminative value of these biomarkers needs to be evaluated across various age ranges, taking into account the differing prevalence of neurodegenerative conditions within specific ages [22].

In this study we analyzed plasma neurodegenerative biomarkers focusing on a large group of cognitively very well-preserved nonagenarians and compared them with younger CU controls and with AD cases. The biomarker profile of this oldest segment of the population is currently unknown and can offer *in vivo* insights into the physiology of brain aging with preserved cognition. Additionally, biomarkers’ levels in this elderly population are important to thoroughly understand their correlation with age and cognitive status across the lifespan, and particularly for pondering the value of NfL and GFAP for the prognosis of clinical dementia.

## MATERIALS AND METHODS

### Participants

We analyzed plasma biomarker results in a cohort of 228 cognitively preserved individuals with available DNA and plasma samples, including 75 nonagenarians and 153 younger CU controls (age range 50 to 85 years), and in 108 AD cases. Demographic data are presented in Table 1. The cohort was ethnic and race homogeneous (white race, South European population). Prior to participating, volunteers, patients or surrogates had provided written informed consent for biological marker studies. The Ethics Committee of Fundación Jiménez Díaz approved this project.

**Table 1:** Population analyzed.

GROUPS	N plasma samples	Age (yrs) Mean ± SD (range)	Gender % female	APOE genotypes
**Nonagenarians CU individuals Young Old AD cases**	75 153 52 101 108	93±3 (90-104) 67±4 (50-70) 75±3 (71-85) 68±6 (51-79)	64% 58% 58% 56%	3/3 (86%); 2/3 (11%); 3/4 (3%) 3/3 (67%); 2/3 (14%); 3/4 (11%); 2/4 (8%) 3/3 (75%); 2/3 (10%); 3/4 (15%) 3/3 (43%); 3/4 (43%); 4/4 (11%); 2/4 (3%)

The group of nonagenarians (n=75, age range 90 to 104 years) had been recruited at Fundación Jiménez Díaz. Preserved cognitive status had been ascertained in all cases by means of interviews with them and their significant others and confirmed with a brief neuropsychological evaluation including MMSE (score ≥ 28/30), Hopkins Verbal Learning Test (score ≥ 7 on the free recall task of 12-item), and Boston Naming Test above the cut-off point for the education level [23]. All nonagenarians were perfectly time-oriented, well aware of current events, and totally independent for basic and instrumental daily living activities.

CU controls (n=153) had been recruited at Alzheimer’s Centre Reina Sofía (n=124; age range 69 to 85, 62% women) from the Vallecas Project, an observational, longitudinal study of elderly volunteers [24], and at the Memory Clinic of Fundación Jiménez Díaz (n=29, age range 50 to 83 years, 38% women). All these participants were yearly assessed with extensive clinical and neuropsychological exams and their cognitive performance was considered normal at baseline and after a follow-up of more than six years in those of the Alzheimer’s Centre Reina Sofía and more than two years in those of the Fundación Jiménez Díaz. Furthermore, 14 of the 29 individuals recruited at Fundación Jiménez Díaz had CSF samples displaying an A-T-N- biomarker profile [1]. This group of 153 CU individuals represented “super” controls, as there were strong guarantees that they were not in a subclinical stage of a neurodegenerative condition. To have a picture of the effect of age on biomarkers’ profiles, CU individuals were allocated into two age groups for analyses: 50 to 70 years (CU young), and 71 to 85 years (CU old). There were no significant differences by sex or APOE genotype between the two groups (Table 1).

The AD comparison group included 108 cases (age mean ± SD = 68 ± 6 years) followed-up in the Memory Clinic of Fundación Jiménez Díaz. Participants were selected from an extensive cohort of demented patients with well-characterized clinical phenotypes with available paired CSF and plasma samples, and with A+T+N+ profile in CSF analysis. The AD biomarkers had been analyzed in CSF using Lumipulse G600II chemiluminescent immunoassay (Fujirebio Iberia, Spain) following the standardized commercial protocol.

### Procedures

EDTA blood samples were centrifuged and plasma aliquots stored at -80°C. Plasma samples were assayed at Alzheimer’s Centre Reina Sofía for: Aβ42, Aβ40, and total tau (T-tau, Neuro3PA kit, Quanterix product code (QPC) 101995), GFAP and NfL (Neuro2PB kit, QPC 103520), and p-tau181 (p-tau181 kit, QPC 104111), using the ultra-sensitive SIMOA- SR-Xtechnology platform (Quanterix, MA) based on previously established methods (www.quanterix.com/SR-X). The experiments were designed to ensure balanced groups comparisons, minimizing bias from inter-assay variability on specific patient groups. However, samples were coded and analyzed blindly.

According to experimental validation parameters, we calculated the concentrations of samples and quality controls (all in pg/ml) using a four-parameter logistic (4PL) curve fit for each biomarker. The calibration range for p-tau181 was 0.0-408 pg/ml, (low quality control range, 31.5- 47.2; high control range 646-968). GFAP calibration ranged from 0.0-7350 pg/ml (lowest control in the 82.4-137 range; highest control range 8812-13219). NfL calibration ranged from 0.0- 7350 pg/ml, the lowest and highest controls were 5.56-8.34 and 439-692, respectively. Finally, the calibration range for Aβ42 was 0.0-31.9 pg/ml (low control 1.59-2.44 and high control 60.8-91.1); for Aβ40, 0.0-111 pg/ml (low control 3.87-6.08 and high control 109-164); and for T-tau 0.0-80.0 pg/ml (low control 1.24-1.85 and high control 53.2-79.7). All curves achieved an R^2^ value above 0.95. Samples were validated in accordance with Quanterix requirements: AEB and fitted concentration coefficient of variation < 0.02.

### Analysis of the data

Biomarker levels in each group were summarized as median and quartiles values. We compared biomarker levels among the groups using the Kruskal-Wallis test and pairwise comparisons between groups were performed using the Mann-Whitney U test applying the Benjamini-Hochberg correction for multiple comparisons. Correlations between plasma biomarker levels and age were analyzed using Spearman coefficients. The differences of biomarkers by sex were evaluated with Mann-Whitney U test, and by *APOE* genotype using Kruskal-Wallis test. Statistical analyses were performed using R version 4.1.2 (R Foundation for Statistical Computing, Vienna, Austria. www.R-project.org/). Figures were designed with Prism 7.0 software.

**Table 2a:** Biomarkers’ concentrations in the studied groups

	CU controls		Nonagenarians	AD cases	Kruskal- Wallis
**Biomarker**	CU young n=52	CU old n=101	n=75	n=108	*P*
**Aβ40 Aβ42 Aβ42/40 ratio T-Tau p-tau181 T-Tau/Aβ42 GFAP NfL**	155 (135, 210) 7.1 (5.7, 8.4) 0.042 (0.037, 0.047) 2.6 (1.9, 3.6) 14 (11, 21) 0.38 (0.28, 0.49) 112 (75, 156) 10 (8, 15)	168 (145, 200) 7.3 (6.2, 8.7) 0.044 (0.039, 0.050) 2.9 (2.0, 3.5) 14 (11, 19) 0.38 (0.28, 0.53) 117 (83, 165) 10 (8, 16)	302 (238, 356) 9.1 (6.9, 11.7) 0.032 (0.027, 0.036) 2.9 (1.8, 4.1) 31 (23, 44) 0.31 (0.19, 0.50) 317 (193, 467) 36 (27, 58)	196 (159, 245) 5.5 (4.4, 7.1) 0.029 (0.027, 0.032) 2.2 (1.6, 3.0) 40 (32, 49) 0.39 (0.28, 0.56) 315 (221, 484) 22 (17, 26)	< 0.001 < 0.001 < 0.001 0.003 < 0.001 0.037 < 0.001 < 0.001

Data represent median (quartiles) in pg/ml.

**Figure 1.**
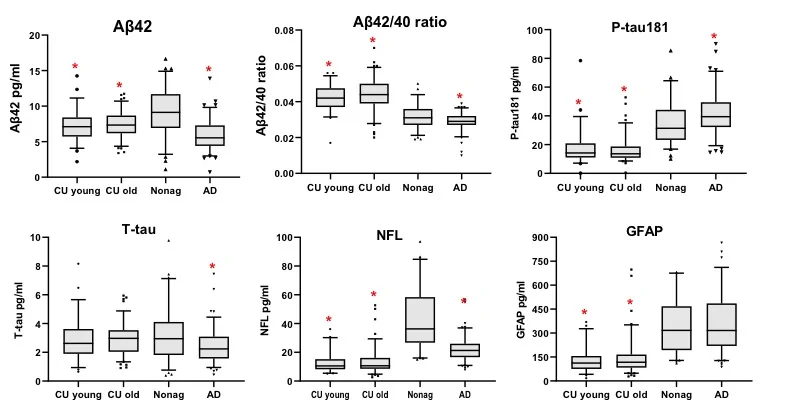
**Box plots of plasma biomarkers by groups**. The boxplots depict the median in the center; the boundaries indicate the first and third quartiles, while the whiskers extend from 5-95 percentile, and the black points indicate individual person values. The red star indicates significant differences of the group versus the Nonagenarians (Mann-Whitney U test applying the Benjamini-Hochberg correction). The number of cases per group are: CU young=52, CU old=101, Nonagenarians=75, AD=108.

## RESULTS

### Nonagenarians compared with CU individuals

Biomarker levels in the three groups of cognitively preserved individuals (young and old CU controls, and nonagenarians) are presented in Table 2a, and boxplots displayed in Figure 1.

Comparison among nonagenarians and CU groups did not show significant differences in T-tau levels and was barely significant in T-tau/Aß42 ratio. In contrast, a consistent pattern was observed in all the other biomarkers (Aß40, Aß42, Aß42/40 ratio, p-tau181, NfL and GFAP): no differences between the CU groups, young versus old, but a very significant difference between each of the CU groups and the nonagenarians (Table 2b). Nonagenarians significantly differed with lower Aß42/40 ratio, higher Aß42, Aß40, and p-tau181 levels, and much higher NfL and GFAP levels (Fig. 1).

**Table 2b:** Statistical comparisons of biomarker levels between groups.

	Comparison between two groups ^§^			
**Biomarker**	CU young vs CU old	Nonag vs CU young	Nonag vs CU old	Nonag vs AD
**Aβ40**	ns	< 0.001	< 0.001	< 0.001
**Aβ42**	ns	< 0.001	< 0.001	< 0.001
**Aβ42/40 ratio**	ns	< 0.001	< 0.001	0.023
**T-tau**	ns	ns	ns	0.016
**p-tau181**	ns	< 0.001	< 0.001	0.001
**T-tau/Aβ42**	ns	ns	0.047	0.047
**GFAP**	ns	< 0.001	< 0.001	ns
**NfL**	ns	< 0.001	< 0.001	< 0.001

* Mann-Whitney test with Benjamini-Hochberg correction. Ns: p >0.05 All comparisons between AD cases and each of the CU groups were significant.

**Table 3:** Correlation of biomarkers with age in cognitively preserved groups.

	CU + Nonag	CU groups	Nonagenarians
**Aβ40**	0.50 (p<0.001)	0.04 (p=0.613)	0.12 (p=0.319)
**Aβ42**	0.29 (p<0.001)	0.04 (p=0.600)	0.07 (p=0.556)
**Aβ42/40 ratio**	-0.46 (p<0.001)	0.09 (p=0.276)	-0.03 (p=0.818)
**T-tau**	0.06 (p=0.402)	0.02 (p=0.843)	0.14 (p=0.268)
**p-tau181**	0.46 (p<0.001)	-0.09 (p=0.292)	0.35 (p=0.006)
**T-tau/Aβ42**	-0.15 (p=0.031)	0.01 (p=0.927)	0.05 (p=0.653)
**GFAP**	0.55 (p<0.001)	0.10 (p=0.250)	0.05 (p=0.722)
**NfL**	0.62 (p<0.001)	0.10 (p=0.236)	0.15 (p=0.201)

Significant correlations are in bold (Spearman coefficients)

When CU controls and nonagenarians were analyzed together, there was a strong correlation of all biomarker levels with age, except for T-tau (Table 3). Aß40 (ρ=0.50, *P*<0.001), Aß42 (0.29, *P*<0.001), p-tau181 (ρ=0.46, *P*<0.001), GFAP (ρ=0.55, *P*<0.001), and NfL (ρ=0.62, *P*<0.001) increased with age, while the Aß42/40 ratio decreased (ρ=-0.46, *P*<0.001). However, these correlations disappeared when analyzing separately the CU groups from the nonagenarians, and only remained significant for p-tau181 when analyzing nonagenarians alone. These data revealed a different profile of biomarkers in subjects aged 50 to 80 years compared to those in their 80s and 90s. In the former group, there is little to no change in slope with age, while in the latter group there is a marked increase in slope.

The distribution of *APOE* genotypes is shown in Table 1. There were no significant effects of APOE genotype on any of the biomarkers when all cognitively preserved individuals were pooled (CU controls and nonagenarians, Table 4), nor when CU controls and nonagenarians were analyzed separately. Most cases were APOE 3/3, and three carriers of APOE 2/4 genotype were not included in the analysis given their low representation. There were no significant differences by gender in any biomarker except for GFAP showing higher levels in women in the CU older group (*P*=0.02).

### Nonagenarians compared with AD cases

There were significant differences in all plasma biomarkers, except GFAP, when compared nonagenarians and AD cases (Table 2b). The nonagenarians showed significantly lower p-tau181 levels (*P*=0.001) and higher Aß42 (*P*<0.001), Aß40 (*P*<0.001), Aß42/40 ratio (*P*=0.023), total tau (*P*=0.02) and NfL (*P*=0.001) than AD cases. Nonagenarians did not reach the pattern of decreased Aß42/40 ratio and increased p-tau181 displayed by the AD cases, but their concentrations were in-between the AD cases and the CU groups (Fig. 1). In addition, GFAP levels were significantly higher in AD cases and nonagenarians compared with CU controls. The strongest discrimination between AD and nonagenarians was obtained by NfL with an AUC=0.85 (95% CI=0.79-0.91) for the cutoff point 32.6 pg/ml, with high sensitivity (94%) but low specificity (64%). Aß42 values higher than 7.8 pg/ml discriminated nonagenarians from AD cases with AUC 0.81 (95% CI=0.73-0.88), also with high sensitivity (87%) but low specificity (68%).

**Table 4:** Biomarker levels in CU groups and nonagenarians according to APOE genotype.

Biomarker	2/3 (n=23)	APOE genotype 3/3 (n=163)	3/4 (n=21)	Kruskal-Wallis	Comparisons by pairs 2/3 vs 2/3 vs 3/3 vs		
				*P*	3/3	3/4	3/4
**Aβ40**	197 (153, 272)	197(154, 277)	161 (137, 222)	0.12	0.99	0.26	0.26
**Aβ42**	7.9 (5.7, 9.7)	7.9 (6.8, 9,9)	6.6 (5.4, 9.0)	0.06	0.50	0.50	0.16
**Aβ42/40 ratio**	0.040 (0.028, 0.048)	0.040 (0.032, 0.046)	0.042 (0.030, 0.045)	0.95	0.97	0.97	0.97
**T-tau**	3.3 (2.3, 3.8)	2.6 (1.8, 3.6)	3.0 (2.3, 3.5)	0.27	0.69	0.89	0.69
**p-tau181**	18 (12, 28)	17 (12, 27)	16 (13, 32)	0.95	1.00	1.00	1.00
**GFAP**	121 (74, 229)	148 (101, 269)	122 (89, 207)	0.24	0.46	0.76	0.61
**NfL**	13.0 (8.7, 30.1)	17.4 (10.0, 32.8)	11.4 (8.0, 25.0)	0.09	0.47	0.49	0.30

Consistent with their diagnosis, AD cases differed from CU controls in a significantly lower Aß42 and Aß42/40 ratio, and a higher p-tau181. In the AD group, only Aß40 had a significant effect of gender, with women exhibiting lower levels than men (p= 0.02). There were no differences in biomarkers levels when compared AD cases younger versus older than 70 years, except for lower levels of Aß40 (*P*=0.001) and Aß42 (*P*=0.016) in the younger group.

## DISCUSSION

This study focuses on characterizing the plasma biomarker profile of a highly selected cohort of cognitively preserved nonagenarians. This investigation is significant as it provides insights into brain aging in cognitively unimpaired and establishes some correlations of neurodegenerative biomarkers in very advanced ages that may have clinical usefulness. The primary finding indicates that cognitively preserved nonagenarians have threefold the level of NfL and GFAP than younger controls, suggesting that neurodegenerative processes are not totally absent from their aging brains. However, they do not develop the AD biomarker signature and display high levels of Aß42.

The examination of AD plasma biomarkers in cognitively preserved nonagenarians offers an opportunity to discern whether these individuals are resistant to AD degeneration (indicated by negative AD biomarkers) or resilient (positive biomarkers). Both scenarios were plausible, as neuropathological studies have documented individuals with abundant Aβ and tau pathology expected to have severe clinical consequences, who nonetheless maintained preserved cognition [25, 26]. Conversely, evidence suggests that it is possible to reach advanced ages without developing AD pathology [27,28].

Our data indicate that the biomarker profile of nonagenarians lacks the characteristic AD signature, thereby suggesting resistance to developing the disease. Notably, this group exhibited the highest levels of Aβ42 and Aβ40, a finding whose significance remains uncertain. The near absence of *APOE*ε4 alleles (only 3% in heterocygosis) and the higher proportion of ε2 alleles in this cohort (11% APOE ε2/ε3 instead of the population-based estimation of 6,8% [29]) may contribute to some extent to both an AD-protective factor and an exceptional longevity. The homozygous status of the ε4 allele has long been known to be associated with a higher risk of developing AD (compared to ε3 alleles), but more recently it is even considered a distinct genetic form of AD [30]. The presence of the ε4 allele has also been reported to increase the risk of cardiovascular disease and reduce the likelihood of reaching exceptional longevity [31,32]. Therefore, the lower prevalence of ε4 alleles in the nonagenarians may account for their longevity together with reduced brain amyloid pathology in this group. However, p-tau181 and Aß42/40 ratio values were found to be at intermediate levels between those of CU controls and AD cases. Given that the CSF Aß42/40 ratio has been identified as the first preclinical change of the AD continuum [21], we could speculate whether these nonagenarians were on the way to incipient amyloidotic changes.

The highest levels of the less specific biomarkers GFAP and NfL exhibited by the nonagenarians (x3 the median of CU controls), suggest the presence of brain astrocytic reactivity and perhaps chronic inflammation and degeneration. GFAP levels were as high as those observed in AD cases, while NfL concentrations were even significantly higher. Both biomarkers, and particularly NfL, have been described to increase with age independently of Aß status [21]. NfL is released into blood and CSF during neuronal damage and is considered a reliable marker to discriminate cases with or without underlying neurodegeneration, with a low false positive rate accross all age-related cutoffs [12]. NfL concentrations do not correlate with Aβ pathology as assessed by amyloid PET [33], and in AD cases they remain more moderate than in other neurodegenerative diseases such as FTD [7,9,34]. Notably, NfL levels can also be influenced by peripheral conditions such as polyneuropathies [35,36]. The very high NfL levels in nonagenarians support the idea that extreme aging is associated with neurodegenerative markers (central and/or potentially peripheral), independent of the presence of Alzheimer’s changes and even in cognitively preserved individuals. These data suggest that in the oldest segment of the population, NfL may lose its value as a marker of central neurodegenerative disease and instead serve as a nonspecific marker of various processes related to aging.

GFAP has been associated with AD as well as with non-AD pathology, such as hippocampal sclerosis [37], common in elderly brains. Several studies have highlighted increased inflammation as a key aspect of brain aging [38,39]. However, interpretations vary on whether this glial activation correlates with brain damage and age-related cognitive decline [40,41], or whether it instead contributes to resilience against aging and AD pathology [42]. Our data suggest that the astrocytic response, as indicated by elevated plasma GFAP levels, may play a protective role against cognitive deterioration, as these levels were very high in cognitively preserved nonagenarians and occurred in the absence of significant markers of AD pathology. A recent study has emphasized the protective role of hippocampal astrocytes during aging in centenarians through the high expression of metallothionein proteins, which have cytoprotective, antioxidant, and anti-inflammatory effects, and are involved in the homeostasis and detoxification of heavy metals [43]. However, the brain’s immune response also involves microglia and a complex variety of cytokine signaling profiles, some of which could be protective while others could be damaging [26,42-46]. On the other hand, blood-brain barrier changes associated with healthy aging [47] may impact fluid biomarker levels as compared with young individuals.

Taken together, the biomarker profile of cognitively preserved nonagenarians suggests that these individuals are quite resilient to aging, having managed to avoid dementia despite high levels of plasma biomarkers associated with neuroinflammation and neurodegeneration changes. Clinically, our results advise caution in the prognosis of clinical dementia based solely on elevated NfL or GFAP values, as these biomarkers may be elevated in elderly individuals with preserved cognition. Prediction of dementia in asymptomatic individuals probably needs additional amyloid and other biomarker information. Thus, we concur with current recommendations that blood-based testing for neurodegeneration should be limited to specialized memory clinics and only for patients exhibiting cognitive symptoms until consensus guidelines are established [22,48].

Lastly, another notable finding from our study is that age-related changes in plasma biomarkers among cognitively healthy individuals begin around the age of eighty, with particularly pronounced increases observed in the nineties. We found a similar range of plasma biomarkers in CU controls aged between 50 and 80 years. These findings contrast with previous studies that have reported a progressive age-related increase in NfL levels [20]. A possible explanation is that our CU sample was highly selected to exclude individuals in subclinical stages of neurodegenerative conditions, as 80% of them were confirmed to remain cognitively preserved after six years of follow-up, and another 10% displayed negative AD biomarkers in CSF. It is also possible that the relationship of biomarkers with age within the range of 60 to 90 years is not linear, with a marked slope over the 80s. However, the number of CU controls in their eighties was small, with a gap between the ages of 86 to 89 years, resulting in an interruption in the age-related slope of the biomarkers.

This study has some limitations: the lack of correlation of the nonagenarians’ plasma biomarkers with other standardized CSF and neuroimaging markers, and the lack of a group of nonagenarians with a definitive neurodegenerative condition of AD type. This information would have provided a more complete picture of this oldest-old population.

In summary, this study reveals a particular plasma biomarker profile in cognitively preserved nonagenarians and underscores the value of these biomarkers as an accessible tool for investigating brain aging in very old populations with limitations for other studies. In addition, the biomarker data reported in this study may be useful in both clinical settings and for future consensus guidelines on plasma biomarker recommendations.
